# The tumor suppressor Zinc finger protein 471 suppresses breast cancer growth and metastasis through inhibiting AKT and Wnt/β-catenin signaling

**DOI:** 10.1186/s13148-020-00959-6

**Published:** 2020-11-17

**Authors:** Chunfang Tao, Juan Luo, Jun Tang, Danfeng Zhou, Shujun Feng, Zhu Qiu, Thomas C. Putti, Tingxiu Xiang, Qiao Tao, Lili Li, Guosheng Ren

**Affiliations:** 1 Key Laboratory of Molecular Oncology and Epigenetics, The First Affiliated Hospital of Chongqing Medical University, Chongqing, China; 2 Hunan Province Key Laboratory of Tumor Cellular and Molecular Pathology, Cancer Research Institute, Hengyang School of Medicine, University of South China, Hengyang, China; 3 Department of Pathology, Yong Loo Lin School of Medicine, National University of Singapore, Singapore, Singapore; 4 Cancer Epigenetics Laboratory, Department of Clinical Oncology, State Key Laboratory of Translational Oncology, Sir YK Pao Center for Cancer and Li Ka Shing Institute of Health Sciences, The Chinese University of Hong Kong and CUHK Shenzhen Research Institute, Hong Kong, China

**Keywords:** ZNF471, CpG methylation, Breast, AKT, Wnt signaling

## Abstract

**Background:**

Zinc-finger protein 471 (ZNF471) is a member of the Krüppel-associated box domain zinc finger protein (KRAB-ZFP) family. *ZNF471* is methylated in squamous cell carcinomas of tongue, stomach and esophageal. However, its role in breast carcinogenesis remains elusive. Here, we studied its expression, functions, and molecular mechanisms in breast cancer.

**Methods:**

We examined ZNF471 expression by RT-PCR and qPCR. Methylation-specific PCR determined its promoter methylation. Its biological functions and related molecular mechanisms were assessed by CCK-8, clonogenicity, wound healing, Transwell, nude mice tumorigenicity, flow cytometry, BrdU-ELISA, immunohistochemistry and Western blot assays.

**Results:**

ZNF471 was significantly downregulated in breast cell lines and tissues due to its promoter CpG methylation, compared with normal mammary epithelial cells and paired surgical-margin tissues. Ectopic expression of ZNF471 substantially inhibited breast tumor cell growth in vitro and in vivo, arrested cell cycle at S phase, and promoted cell apoptosis, as well as suppressed metastasis. Further knockdown of ZNF471 verified its tumor-suppressive effects. We also found that ZNF471 exerted its tumor-suppressive functions through suppressing epithelial-mesenchymal transition, tumor cell stemness and AKT and Wnt/β-catenin signaling.

**Conclusions:**

ZNF471 functions as a tumor suppressor that was epigenetically inactivated in breast cancer. Its inhibition of AKT and Wnt/β-catenin signaling pathways is one of the mechanisms underlying its anti-cancer effects.

## Introduction

In women, breast cancer is the most commonly diagnosed cancer and the leading cause of cancer death worldwide [1, 2]. It is a heterogeneous disease divided into five major subtypes by DNA microarray gene expression profiling: luminal A (estrogen receptor [ER]+, progesterone receptor [PR]±, HER2−), luminal B (ER+ , PR± , HER2+), HER2-enriched (ER−, PR−, HER2 +), basal-like (ER−, PR−, HER2−), and normal-like cancers [3, 4]. Currently, the main treatment methods for breast cancer are surgery, medical oncology (chemotherapy, endocrine therapy, or HER2-directed therapy), and radiation. Endocrine therapy and HER2 targeted therapies have led to a dramatic improvement in the overall prognosis of ER/PR+ and HER2+ subtypes, respectively [5]. However, the incidence and mortality of breast cancer remain high in many countries. Therefore, understanding the molecular mechanisms involved in breast cancer development is critical.

The Wnt/β-catenin pathway participates in the development of various tumors. Wnt signaling can facilitate tumor metastasis [6] and is important for maintaining cell stemness [7–9]. Activation of the Wnt pathway can be detected in more than half of breast cancers, and patients with Wnt pathway activation tend to have worse clinical outcomes [10]. The AKT pathway has been shown to be involved in breast cancer cell proliferation, apoptosis, and metastasis [11, 12].

Cancer is driven by accumulated genetic and epigenetic alterations. CpG island hypermethylation, a common epigenetic change, has been found in almost all types of human tumors [13]; thus, methylation might be an ideal candidate for early detection of tumors [14]. In breast cancer, promoter hypermethylation has been reported to be involved in tumor suppressor gene (TSG) silencing [15–17].

Krüppel-associated box domain zinc finger proteins (KRAB-ZFPs) are the largest family of transcriptional regulators in mammals and are characterized by the presence of a C-terminal array of a DNA-binding domain and an N-terminal KRAB domain [18]. KRAB-ZFPs have been implicated in cell differentiation, proliferation, apoptosis, neoplastic transformation, and metabolic pathways [18, 19]. KRAB-ZFPs have also been linked to cancer development [20–23]. ZNF471 is a member of the KRAB-ZFP family, located in 19q13.43. It has been identified as a TSG in gastric cancer where it directly inhibits transcription of its downstream targets TFAP2A and PLS3 [24]. In esophageal cancer, ZNF471 functions as a TSG through activating MAPK10/JNK3 signaling [25]. However, the role of ZNF471 in breast cancer remains unclear. In this study, we showed that ZNF471 was silenced or downregulated in breast cancer due to promoter methylation. We also showed that ZNF471 exerted its tumor-suppressive functions through AKT/GSK-3β and Wnt/β-catenin signaling pathways.

## Results

### *ZNF471* downregulation in breast cancer is associated with poor patient survival

**Fig. 1:**
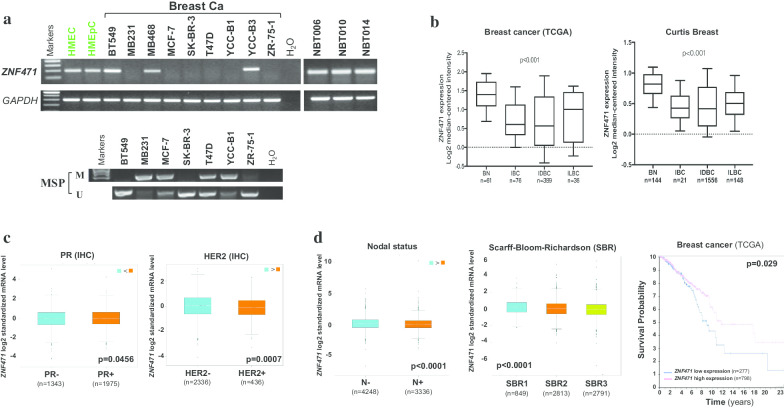
Expression of ZNF471 in breast cancer and its association with clinicopathologic features of breast tumors and survival probability. **a** ZNF471 expression levels were examined in nine breast cancer cells, normal mammary epithelial cell lines and normal breast tissues by RT-PCR, NBT: normal breast tissue. The promoter methylation levels of ZNF471 in breast cancer cells. **b** ZNF471 expression in breast cancer from the Oncomine database (https://www.oncomine.org/). IBC: Invasive Breast Carcinoma; IDBC: Invasive Ductal Breast Carcinoma; ILBC: Invasive Lobular Breast Carcinoma (ILBC). **c** ZNF471 mRNA expression was associated with PR, HER2 status in breast cancer. (https://bcgenex.centregauducheau.fr/BC-GEM/GEM-Accueil.php?js=1). **d** ZNF471 mRNA expression was related to lymph node metastasis, SBR grade and survival probability in breast cancer

To assess whether ZNF471 is downregulated in breast tumors, we first examined the expression of ZNF471 in a panel of breast cancer cell lines, normal mammary epithelial cell lines (HMEC and HMEpC) and normal breast tissues by semiquantitative RT-PCR. ZNF471 was readily detected in HMEpC and HMEC cells, but dramatically reduced or silenced in six of nine breast cancer cell lines, (Fig. 1a). Data from the Oncomine database (https://www.oncomine.org/) showed that *ZNF471* mRNA expression was downregulated in Invasive Breast Carcinoma (IBC), Invasive Ductal Breast Carcinoma (IDBC) and Invasive Lobular Breast Carcinoma (ILBC) compared to normal breast tissues (Fig. 1b). Furthermore, ZNF471 expression was associated with progesterone receptor (PR), HER2, nodal status and tumor grade of breast cancer. These data indicated that *ZNF471* expression is frequently downregulated in breast cancer and associated with clinicopathologic features including PR, HER2 status, lymph node metastasis and higher histologic grade (Fig. 1c, d). To analyze the relationship between ZNF471 and survival in breast cancer, a prognostic analysis was next performed using the Human Protein Atlas database (https://www.proteinatlas.org/). Results showed that patients with higher ZNF471 mRNA expression levels had increased survival probability compared to those with low ZNF471 mRNA levels (Fig. 1d). We further performed the univariate and multivariate Cox regress analyses through analyzing breast cancer genomic data from the TCGA database (*n* = 639), however,we found that ZNF471 is not an independent predictor in breast cancer (Additional file 1: Table S1). The difference between our result and the Human Protein Atlas database result may be due to the different samples selected.

To analyze the relationship between ZNF471 and survival in breast cancer, a prognostic analysis was next performed using the Human Protein Atlas database (https://www.proteinatlas.org/). Results showed that patients with higher ZNF471 mRNA expression levels had increased survival probability compared to those with low ZNF471 mRNA levels (Fig. 1d).

### Promoter methylation mediates* ZNF471* downregulation in breast cancer

**Fig. 2:**
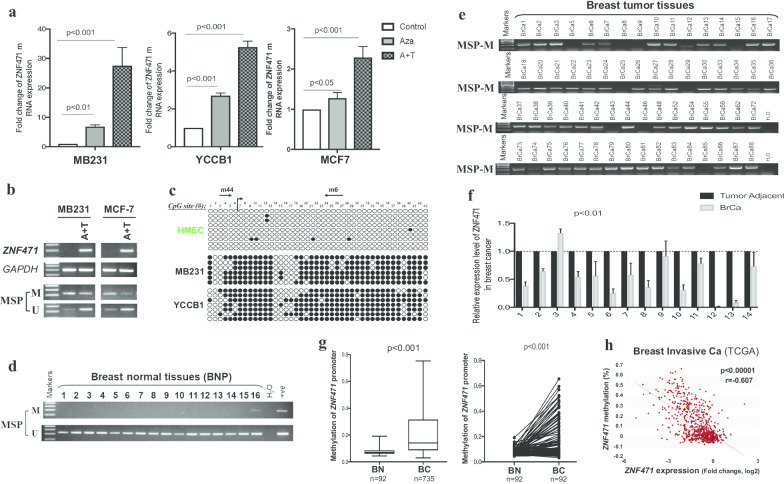
ZNF471 is downregulated in breast cancer cell lines and tissues due to promoter methylation. **a**, **b** Pharmacological demethylation restored the expression of ZNF471 in breast cancer cell lines, with demethylation of the promoter. M, methylated; U, unmethylated. **c** High-resolution methylation analysis of ZNF471 promoter by BGS in HMEC, MB231 and YCCB1 cells. ZNF471 promoter methylation levels were detected in breast normal tissues (**d**) and breast cancer tissues (**e**). **f** ZNF471 mRNA expression in primary breast tumor tissues (*n* = 14) and paired adjacent non-cancerous tissues (*n* = 14) by qPCR. **g** ZNF471 promoter methylation in breast cancer tissues and normal breast tissues from an online database (https://methhc.mbc.nctu.edu.tw/). BN: breast normal tissues; BC: breast cancer tissues. **h** An online database was used to analyze the correlation between the expression of ZNF471 and its promoter methylation in breast cancer (https://methhc.mbc.nctu.edu.tw/). Error bars mean standard deviation (SD); values are presented as the mean ± SD of at least three independent experiments (*p < 0.05; **p < 0.01; ***p < 0.001)

We next examined whether ZNF471 downregulation in breast cancer was due to promoter methylation. ZNF471 was methylated in 4 of 7 breast cancer cell lines (Fig. 1a). A pharmacological demethylation experiment was performed in which MDA-MB-231, YCC-B1 and MCF-7 cells were treated with the DNA methyltransferase inhibitor 5-aza-2′-deoxycytidine (Aza) alone or in combination with the HDAC inhibitor trichostatin A (TSA). The results indicated that pharmacologic demethylation partially restored the expression of ZNF471, along with decreased methylated alleles and increased unmethylated alleles as detected by methylation-specific PCR (MSP) (Fig. 2a, b). High-resolution bisulfite genomic sequencing (BGS) analysis was performed to examine the methylation status of 43 individual CpG sites within the ZNF471 promoter CGI, with a higher density of methylated alleles were observed in methylated MB231 and YCCB1 cell lines compared with HMEC cell lines, consistent with the MSP results (Fig. 2c).

Moreover, ZNF471 methylation was detected in 53/64 (82.8%) tumors but only 1/16 (6.25%) in breast normal tissues (Fig. 2d, e; Additional file 2: Fig. S1). Consistently, the mRNA level of ZNF471 was significantly lower in 12 of 14 pairs of breast cancer tissues compared with paired surgical-margins (Fig. 2f). Online databases were further used to investigate whether *ZNF471* downregulation in breast cancer was related to promoter methylation (https://methhc.mbc.nctu.edu.tw/). Results showed that *ZNF471* methylation was far more prevalent in breast cancer tissues than in normal breast tissues, and downregulation of ZNF471 in breast cancer was significantly inversely correlated with its methylation (Fig. 2g, h). These data indicated that *ZNF471* was downregulated in breast cancer due to promoter methylation.

### ZNF471 inhibits breast tumor cell growth and colony formation

**Fig. 3:**
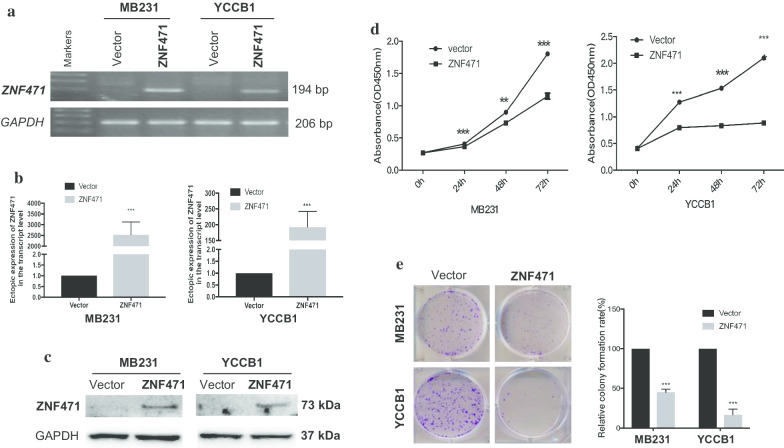
ZNF471 inhibits breast cancer cell growth and colony formation. Ectopic expression of ZNF471 in MB231 and YCCB1 cells was measured by RT-PCR (**a**), qRT-PCR (**b**) and Western blot assay (**c**). **d** Cell viabilities were measured with CCK-8 assay at 24, 48 and 72 h in vector- and ZNF471-stably transfected MB231 and YCCB1 cells. **e** Cell colony formation ability was evaluated by clonogenicity in vector- and ZNF471-expressing MB231 and YCCB1 cells. Left: Representative images. Right: Data summary. All values are presented as the mean ± SD of at least three independent experiments. SD, standard deviation. **p < 0.01; ***p < 0.001

To clarify the effect of ZNF471 in breast cancer, we first established cell lines that stably overexpress ZNF471. MDA-MB-231 and YCC-B1 cells were transfected with empty pcDNA3.1 and ZNF471 and selected with G418 for 14 days. Expression levels of ZNF471 in the transfected cells were examined by RT-PCR, qPCR, and western blotting (Fig. 3a–c). Colony formation and CCK-8 proliferation assays were performed to assess the effect of ZNF471 on cell proliferation in breast cancer. The CCK-8 assay showed that cell viability was decreased at 24, 48, and 72 h in ZNF471-expressing cells (Fig. 3d). A reduction of approximately 55–80% in colony formation was observed in ZNF471-expressing cells compared to control cells (Fig. 3e). These results indicated that ZNF471 suppressed cell colony growth and colony formation in breast cancer.

### ZNF471 induces S cell cycle arrest and induces apoptosis in breast cancer

**Fig. 4:**
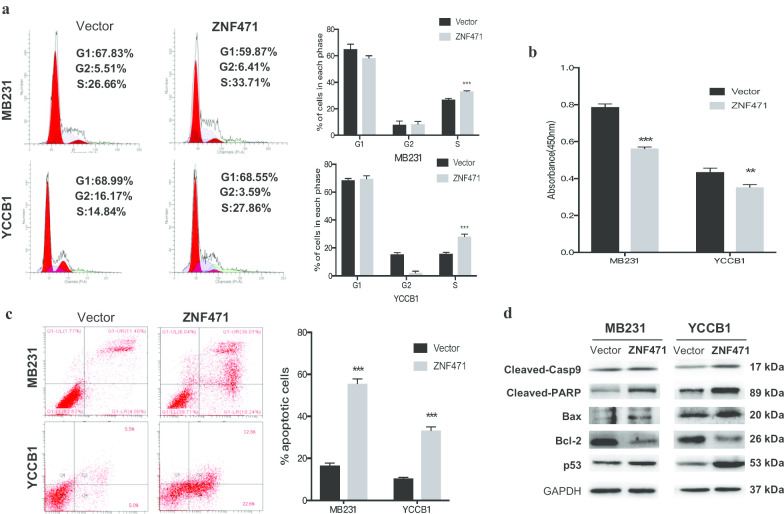
ZNF471 induces cell cycle at S phase and induces apoptosis. **a** The cell cycle distribution was measured by flow cytometry analysis in vector- and ZNF471-expressing MB231 and YCCB1 cells. Left: Representative flow cytometry plots. Right: Data summary. **b** BrdU-ELISA assay at 24 h in vector- and ZNF471-transfected MB231 and YCCB1 cells. **c** The percentages of apoptotic cells were evaluated in vector- and ZNF471-transfected MB231 and YCCB1 cells. Left: Representative flow cytometry plots. Right: Quantitative analysis. **d** Western blot analysis of apoptotic markers (cleaved-caspase 9, cleaved-PARP, Bax, Bcl-2, and p53). All values are presented as the mean ± SD of at least three independent experiments. SD, standard deviation. **p < 0.01; ***p < 0.001

We next examined the role of ZNF471 in cell cycle distribution and apoptosis via flow cytometry. There was an increase in the number of S phase cells in ZNF471-transfected MD-MB-231 and YCC-B1 cells compared to empty-transfected cells (Fig. 4a). Furthermore, the BrdU-ELISA assay, which reflects active DNA synthesis, revealed that cell proliferation was decreased in ZNF471-expressing cells (Fig. 4b). Flow cytometric analysis also revealed that ZNF471 overexpression dramatically induced apoptosis compared to the control (Fig. 4c). We next assessed the expression of the following apoptotic markers by western blotting: cleaved-caspase 9, cleaved-PARP, Bax, Bcl-2, and p53. Expression of cleaved-caspase 9, cleaved-PARP, Bax and p53 was increased, while the expression of Bcl-2 was decreased, further confirming the effect of ZNF471 on apoptosis (Fig. 4d). Taken together, these data showed that ZNF471 blocked the cell cycle in the S phase and inducing apoptosis.

### ZNF471 suppresses breast tumor cell migration and invasion

**Fig. 5:**
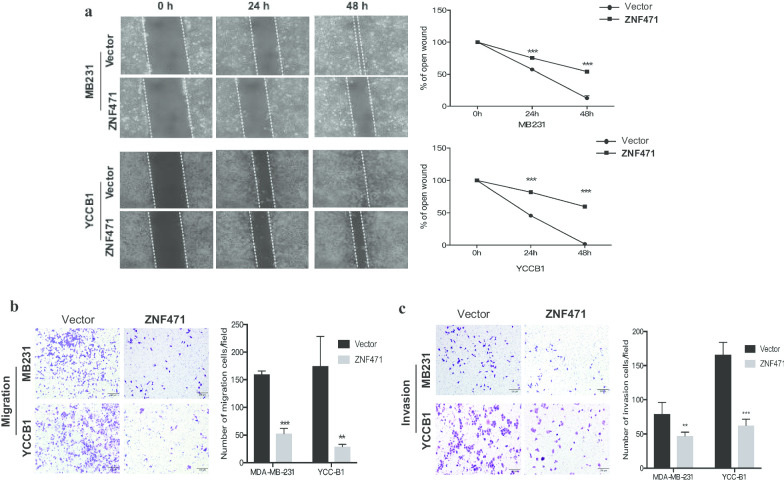
ZNF471 suppresses migration and invasion of breast tumor cells. **a** Wound healing assay in vector- and ZNF471-transfected MB231 and YCCB1 cells at 0, 24, and 48 h. Left: Representative images. Right: Quantitative analysis of the wound healing rate. **b** Transwell assay showed the migration ability of vector- and ZNF471-expressing MB231 and YCCB1 cells Left: Representative images. Right: Data summary. **c** Transwell assay showed the invasion ability of vector- and ZNF471-expressing MB231 and YCCB1 cells. Left: Representative images. Right: Data summary. Data are presented as the mean ± SD. **p < 0.01; ***p < 0.001

To evaluate the effect of ZNF471 on cell migration and invasion, wound healing and Transwell assays were performed. The wound-healing assay showed that the rate of healing in ZNF471-overexpressing cells was significantly slower than that in control cells at 24 and 48 h (Fig. 5a). Furthermore, the number of ZNF471-overexpressing cells that passed through the chamber membrane was decreased compared to cells transfected with the empty control vector in the Transwell migration assay (Fig. 5b). These data demonstrated that ZNF471 inhibited breast cancer cell migration. A Matrigel invasion assay revealed that the number of cells passing through the chamber membrane with a Matrigel barrier was lower than control cells (Fig. 5c). Thus, ZNF471 can suppress cell invasion. These results indicated that ZNF471 inhibits cell migration and invasion.

### Knockdown of ZNF471 promotes cell growth, induces metastasis and inhibits apoptosis

**Fig. 6:**
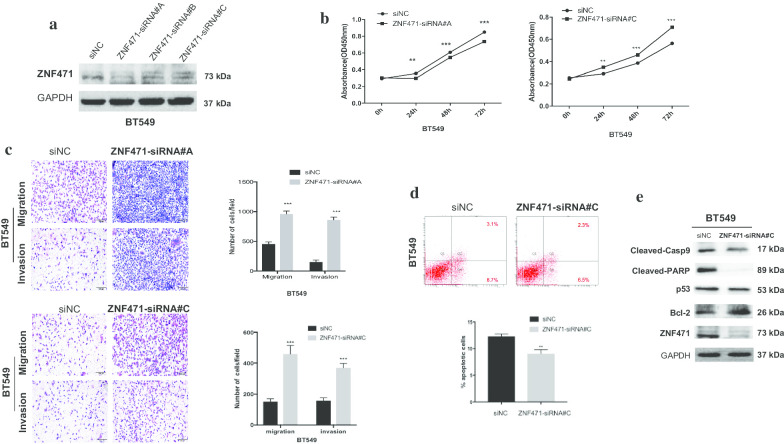
Knockdown of ZNF471 promotes cell growth, metastasis, and inhibits cell apoptosis. **a** Western blot assay of ZNF471 knockdown efficiency in BT549 cells after transfected with. ZNF471. siRNA and control siRNA. **b** Cell viabilities were measured by CCK-8 assay at 24, 48 and 72 h in BT549 cells after ZNF471 knockdown. **c** Transwell assay showed cell migration and invasion of BT549 cells after the ZNF471 knockdown. Left: Representative images. Right: Data summary. **d** Flow cytometry assay of relative cell apoptosis rate in BT549 cells after transfected with ZNF471 siRNA and control siRNA. (E) Western blotting analyzed the expression of cleaved-casppase9, cleaved PARP, Bcl-2, and p53 in ZNF471 knockdown cells. Data are presented as the mean ± SD. **p < 0.01; ***p < 0.001

To further demonstrate the tumor suppression function of ZNF471 in breast cancer, we knockdown of ZNF471 in ZNF471-expressing breast cancer cell line BT549 by siRNA transfection. Western blot assay was used to detect the knockdown efficiency of ZNF471 (Fig. 6a). In the CCK-8 assay, we found that cell viability was increased after ZNF471 knocking down (Fig. 6b). Cell migration and invasion abilities were measured by Transwell assays, results indicated that knockdown of ZNF471 induced cell migration and invasion compared with cells transfected with control siRNA (Fig. 6c). Moreover, flow cytometry assay and western blotting demonstrated that knockdown of ZNF471 inhibited cell apoptosis (Fig. 6d, e). These knockdown data confirmed that ZNF471 has a tumor-suppressive effect on breast cancer.

### ZNF471 attenuates the epithelial-mesenchymal transition (EMT) and suppresses cell stemness in breast cancer

**Fig. 7:**
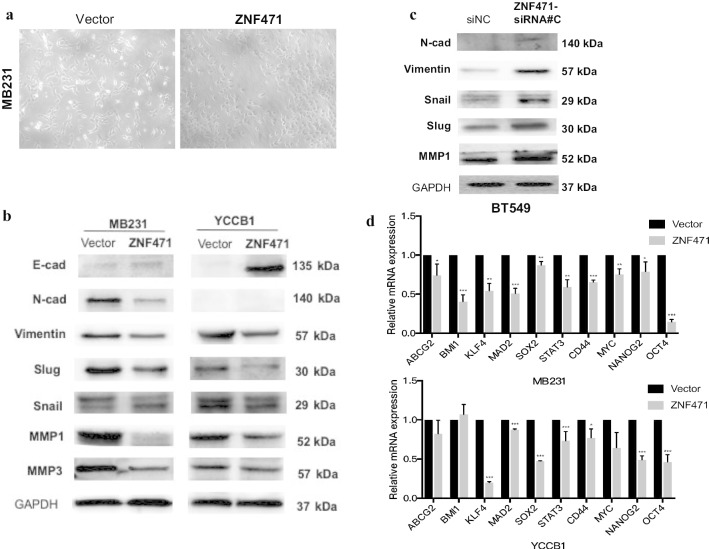
ZNF471 attenuates the epithelial-mesenchymal transition (EMT) and suppresses cell stemness in breast cancer. **a** Morphological changes of MB231 cells after transfected with vector and ZNF471. **b** Expression of E-cadherin, N-cadherin, Vimentin, Snail, Slug, and MMP1,3 by western blot assay in empty vector cells and ZNF471 overexpressing cells. **c** Western blot was performed to examined N-cad, vimentin, slug, snail and MMP1 in BT549 cells. **d** qPCR analysis of stem cell markers expression (Data are presented as the mean ± SD; *p < 0.05; **p < 0.01; ***p < 0.001)

Because the EMT plays an important role in cell migration and invasion by reducing cell–cell adhesion and increasing motility, we first tested whether ZNF471 inhibits breast cancer cell migration and invasion by attenuating the EMT. Morphological changes of cells stably expressing ZNF471 and empty vector cells were observed by phase-contrast microscopy. We found that the morphology of ZNF471-expressing cells dramatically changed from a spindle-like morphology to a typical cobblestone-like morphology (Fig. 7a). Western blot was then performed to detect the expression of EMT markers, the results revealed that the epithelial marker E-cadherin was upregulated while Vimentin, Snail, Slug, and N-cadherin were downregulated. Metastasis markers matrix metalloproteinase MMP1 and MMP3 were also downregulated in ZNF471-overexpressing cells (Fig. 7b). However, the expression of EMT markers and MMP1 was upregulated after ZNF471 knocking down (Fig. 7c). Thus, ZNF471 suppressed breast cancer cell metastasis by attenuating the EMT.

As the EMT is closely related to cancer cell stemness, we next verified whether ZNF471 affects cell stemness. We analyzed stem cell markers (*ABCG2, BMI1, KLF4, MAD2, STAT3, CD44, MYC, NANOG2*, and *OCT4*) by qPCR. We found that expression of ZNF471 resulted in the downregulation of almost all of the stem cell markers in MDA-MB-231 and YCC-B1 cells (Fig. 7d). Thus, ZNF471 suppressed cell stemness in breast cancer.

### ZNF471 inhibits breast tumor growth in vivo

**Fig. 8:**
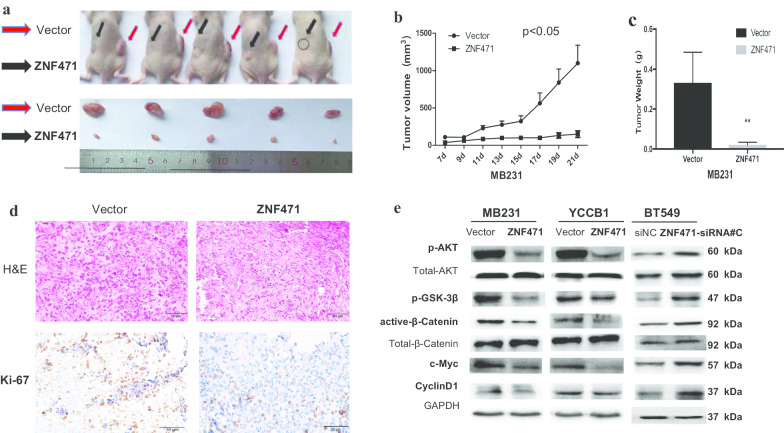
ZNF471 inhibits breast tumor growth in vivo. ZNF471 exerts its tumor-suppressive function by blocking AKT and Wnt/β-catenin signaling pathways. **a** Images of human breast tumor xenografts. **b** Tumor growth curve for vector- and ZNF471-expressing MB231 cells in nude mice xenografts. **c** Tumor weights in the two groups of nude mice. **d** Representative photographs of H&E staining and IHC expression analyses of Ki-67. **e** Expression of phospho-AKT, phospho-GSK-3β, active-β-catenin, and the following downstream target genes: c-Myc, cyclin D1 in MB231, YCCB1 and BT549 cells. Data are presented as the mean ± SD. *p < 0.05; **p < 0.01

Given that ZNF471 suppressed breast cancer cell proliferation in vitro, we further tested its function in vivo. Stable MDA-MB-231 cells transfected with ZNF471 and empty pcDNA3.1 were injected into nude mice. Tumor length and width were measured every 2 days. Tumors were derived from mice after 21 days for further analysis. Tumor volume (length × width^2^) and average weight were significantly lower in cells with stable expression of ZNF471 than that in control tumors (Fig. 8a–c). Immunohistochemistry and hematoxylin and eosin-stained sections showed that expression of Ki-67, a marker of cell proliferation, was significantly decreased, and the number of tumor cells exhibiting nuclear fragmentation was increased (Fig. 8d). These results suggest that ZNF471 inhibits breast cancer growth in vivo.

### ZNF471 exerts its tumor-suppressive function by blocking AKT and Wnt/β-catenin signaling pathways

AKT and Wnt/β-catenin pathways have been reported to be associated with breast cancer cell proliferation and metastasis. We assumed that the anti-cancer effect of ZNF471 in breast cancer was achieved by modulating AKT and Wnt/β-catenin signaling. Therefore, expression levels of key proteins of the AKT and Wnt/β-catenin pathways were investigated by western blotting. The results showed that ectopic expression of ZNF471 downregulated expression of phospho-AKT, phospho-GSK-3β, active-β-catenin, and the following downstream target genes: c-Myc, cyclin D1 (Fig. 8e). In contrast, knockdown of ZNF471 in BT549 cells by siRNA upregulated expression of them. Collectively, ZNF471 was a negative regulator of AKT and Wnt/β-catenin pathways and exerted its anti-cancer effect by inhibiting the activation of AKT and Wnt/β-catenin signaling.

## Methods and materials

### Tumor cell lines and tissues

A panel of breast cancer cell lines (MDA-MB-231, MDA-MB-468, BT549, MCF-7, T47D, SK-BR-3, YYC-B1, YCC- B3, and ZR-75–1) was used. Human mammary epithelial cell lines HMEpC and HMEC were used as controls. All cell lines were maintained at 37 °C in RPMI 1640 with 10% fetal bovine serum (FBS; Invitrogen, Carlsbad, CA, USA). Human normal adult breast tissue RNA samples were purchased commercially (Stratagene, La Jolla, CA, USA; Millipore Chemicon, Billerica, MA, USA; and BioChain Institute, Hayward, CA, USA). Breast cancer tissues and paired surgical-margin tissues were obtained from the First Affiliated Hospital of Chongqing Medical University. This study was approved by the ethics committee of the first affiliated hospital of Chongqing Medical University, and all patients signed consent forms.

### RNA isolation, reverse transcription-PCR, semiquantitative RT-PCR, and quantitative real-time PCR

Total RNA was extracted from cell lines and tissues using TRIzol reagent (Invitrogen). RNA was reverse-transcribed according to the instructions of the Reverse Transcription System (Promega, Madison, WI, USA). RT-PCR was carried out with Go-Taq DNA polymerase (Promega) and performed using a final volume of 10 μL reaction mixture containing 2 μL cDNA for 32 cycles of amplification as previously reported [26]. 2% agarose gels were used to assay amplified PCR products. GAPDH served as a control. The qPCR was carried out by Maxima SYBR Green/ROX qPCR MasterMix (MBI Fermentas, St. Leon-Rot, Germany) on an Applied Biosystems 7500 Real-Time PCR System (Applied Biosystems, Foster City, CA)according to the manufacturer’s instructions [26]. The relative expression of ZNF471 was calculated by the 2(− ΔCt) method. GAPDH was used as a control. All samples were assessed in triplicate. All primers are shown in Table 1.

**Table 1:** List of primers used in this study

PCR	Primer	Sequence (5′-3′)	Product Size (bp)	PCR cycles	Annealing temperature (°C)
RT-PCR	ZNF471F	GAGATGACGAGTGAGATGAC	194	32	55
	ZNF471R	TGACTTCCCATCTGCTTCTC			
	GAPDHF	GGAGTCAACGGATTTGGT	206	23	55
	GAPDHR	GTGATGGGATTTCCATTGAT			
qPCR	ZNF471F	GAGATGACGAGTGAGATGAC	194		60
	ZNF471R	TGACTTCCCATCTGCTTCTC			
	GAPDHF	CCAGCAAGAGCACAAGAGGAA	114		60
	GAPDHR	GGTCTACATGGCAACTCAAGG			
MSP	ZNF471m44	TTTTGTTTTCGTTTTTTTCGTTC	223		60
	ZNF471m6	ACGCGACTAAACCTTCGCG			
	ZNF471u44	GTTTTGTTTTTGTTTTTTTTGTTT	228		58
	ZNF471u6	AAAAACACAACTAAACCTTCACA			
BGS	ZNF471BGS1	GGTTTTTTTTATTTTTTTAGTAGTT	410	41	60
	ZNF471BGS4	TCCCACAACTCACTCCAATAA			

### Methylation-specific PCR and bisulfite genomic sequencing

To evaluate the methylation of ZNF471, bisulfite modification of DNA, MSP and BGS were performed as previously [27, 28]. Genomic DNA was isolated from tissues and cell lines using a QIAamp DNA Mini kit (Qiagen, Hilden, Germany).MSP was carried out using AmpliTaq-Gold DNA Polymerase (Applied Biosystems) with the following conditions: 95 °C for 10 min and 40 3-step cycles (94 °C for 30 s, 58 °C for 30 s and 72 °C for 30 s), a final extension of 5 min at 72 °C. PCR products were assessed with 2% agarose gels. The primers are listed in Table 1.

### 5-Aza-2′-deoxycytidine and trichostatin A treatment

Cell lines were treated with 10 mM Aza (Sigma-Aldrich, St. Louis, MO, USA) for 3 days followed by treatment with or without 100 nM TSA (Cayman Chemical Co., Ann Arbor, MI, USA) for 24 h. Cells were then collected for RNA extractions.

### Construction of vector- and ZNF471-expressing stable cell lines

MDA-MB-231 and YCC-B1 cells were plated in six-well plates and transfected with pcDNA3.1 and pcDNA3.1–ZNF471 plasmids at a concentration of 4 µg using Lipofectamine 2000 (Invitrogen). At 48 h after transfection, G418 was added to screen cells expressing ZNF471 for 14 days. Stable cells were further verified by RT-PCR, qPCR, and western blotting.

### Cell viability assays

Vector- and ZNF471-expressing cells were plated in 96-well plates at a density of 2000 cells/well. After the cells were adherent, Cell Counting Kit-8 (CCK-8; Dojindo Molecular Technologies, Inc., Kumamoto, Japan) reagent was added at a concentration of 10 μL/100 μL complete medium to assess viability at 0, 24, 48, and 72 h. Absorbance was read in a microplate reader at 450 nm. All experiments were performed in triplicate.

### Colony formation assay

Stably expressing ZNF471 and empty vector cells were seeded in six-well plates at 200, 400, or 800 cells/well in complete medium containing G418. Surviving colonies (> 50 cells per colony) were counted after 10–14 days, fixed with 4% paraformaldehyde, and stained with crystal violet. All experiments were performed in triplicate.

### Flow cytometry

Flow cytometry was performed to evaluate the cell cycle and apoptosis. For cell cycle analysis, cells stably expressing ZNF471 and vector control cells were trypsinized with pancreatin, washed once with phosphate-buffered saline (PBS), and fixed in 70% ice-cold ethanol overnight. Propidium iodide (PI) was used to stain cells for 30 min in the dark. For apoptosis, cells were transfected with pcDNA3.1 and pcDNA3.1–ZNF471 plasmids at a concentration of 4 μg using Lipofectamine 2000 (Invitrogen). After 48 h, cells were trypsinized with pancreatin, washed once with PBS, and incubated with Annexin V-fluorescein isothiocyanate (FITC; BD Pharmingen) and PI (Sigma-Aldrich) at room temperature for 30 min. The flow cytometry results were evaluated using a FACSCalibur machine (BD Biosciences, Franklin Lakes, NJ, USA). Experiments were performed in triplicate.

### BrdU cell proliferation enzyme-linked immunosorbent assay

The BrdU Cell Proliferation ELISA Kit (Abcam, Cambridge, UK) was used for the detection of DNA synthesis and cell proliferation. Following transfection with ZNF471 and pcDNA3.1 plasmids for 48 h, cells were plated in 96-well plates at 1 × 105 cells/mL in 100 μL/well of complete cell culture media. After 24 h of culture, bromodeoxyuridine (BrdU) was added into the wells for another 12 h. The BrdU-ELISA assay was performed according to the manufacturer’s instructions. Results were read using a microplate reader at 450 nm.

### Wound healing, migration, and Matrigel invasion assays

Stably-expressing ZNF471 and empty vector cells were plated in 6-well plates and cultured overnight until confluent. Then, the cell monolayer was scratched with a sterile pipette tip, and the cells were washed once with PBS and cultured in serum-free medium. Cell migration was observed under a phase-contrast microscope (Leica DMI4000B, Milton Keynes, Bucks, UK) every 24 h. All experiments were performed in triplicate.

Transwell migration and invasion assays were performed to assess cell migration and invasion. Transwell chambers (8-µm pore size; Corning, Inc., Corning, NY, USA) were used. The cells were treated with serum starvation overnight in the Transwell migration and invasion assays. Cells were plated in the upper chamber at 2 × 10^4^ cells/chamber in 200 μL serum-free medium, with 700 μL medium containing 10% FBS in the lower chamber. A Matrigel (BD Biosciences) barrier was added on top of the Transwell membrane to verify cell invasiveness. After incubation for 24 h, cells were fixed with 4% paraformaldehyde for 30 min and stained with crystal violet for 20 min. Cells were counted using microscopy. All experiments were performed in triplicate.

### Western blot

Cellular proteins were extracted and western blotting was performed as previously described [26, 29, 30]. Protein lysates (40 μg) were separated by sodium dodecyl sulfate‐polyacrylamide gel electrophoresis and transferred onto polyvinylidene fluoride membranes. Blocking was performed using 5% milk for 1 h at room temperature. The following primary antibodies were used: ZNF471 (ab204974; Abcam), total AKT (#4691; Cell Signaling Technology, Danvers, MA, USA), phospho‐AKT (#13038; Cell Signaling Technology), total β-catenin (#9562; Cell Signaling Technology), active β-catenin (#4272; Cell Signaling Technology), p-GSK-3β (sc-373800), c‐Myc (#13987; Cell signaling Technology), cyclin D1 (sc-450; Santa Cruz Biotechnology), vimentin (sc‐6260; Santa Cruz Biotechnology), E‐cadherin (sc‐8426; Santa Cruz Biotechnology), N-cadherin (#14215; Cell Signaling Technology), Snail (#3879; Cell Signaling Technology), Slug (sc-166476; Santa Cruz Biotechnology), cleaved caspase 9 (WL01838; Wanleibio, Shenyang, China), Bcl2 (#2870; Cell Signaling Technology), Bax (#502; Cell Signaling Technology), cleaved-PARP (#7851; Cell Signaling Technology), p53 (sc-47698, Santa Cruz Biotechnology), MMP1/8 (sc-137044, Santa Cruz Biotechnology), and MMP3 (sc-21732; Santa Cruz Biotechnology). Proteins were imaged using an enhanced chemiluminescence detection system (Pierce Chemical Co., Rockford, IL, USA). GAPDH was used as the protein expression control.

### Small interfering RNA (siRNA)

ZNF471 siRNA kits were purchased from OriGene ( OriGene Technologies, Rockville, MD). Transfections were performed using Lipofectamine 2000 according to the manufacturer’s instructions with a concentration of 10 nM siRNA. The cells were collected for RNA or protein extraction at 48–72 h after transfection.

### In vivo tumorigenicity

To verify the inhibitory effect of ZNF471 on tumors in vivo, a nude mice tumorigenicity assay was performed. Female BALB/c nude mice (aged 4–6 weeks) were obtained from the Experimental Animal Center of Chongqing Medical University (CQMU; China). Stable ZNF471-expressing MDA-MB-231 cells or empty control cells (2.5 × 10^6^ cells in 0.2 mL PBS) were injected into the left and right backs of nude mice. 21 days after injection, tumors were derived from the mice and its size and average weight were analyzed. Tumors were then fixed, dehydrated, and embedded in paraffin for immunohistochemistry and hematoxylin and eosin staining. Animal experiments were approved by the ethics committee of CQMU.

### Immunohistochemistry staining

Immunohistochemistry was performed as described previously [17, 31]. Briefly, the tumor sections of nude mice were dewaxed, rinsed, rehydrated, and antigen repaired. The sections were then incubated overnight at 4 °C with anti-Ki67 (1:400, ab15580; Abcam) antibody, followed by secondary antibody at 37 °C for 30 min. Diaminobenzidine (DAB) was used for color development and hematoxylin was used for counterstaining. Images were captured under a microscope at 400 × magnification.

### Statistical analysis

For statistical analysis, Graphprism 7.0 (La Jolla, California) and SPSS23.0 software (Chicago, IL) were used. All experimental analysis was performed in triplicate, and results are expressed as the mean ± SD compared by Student’s t-test. Univariate and multivariate Cox regression analyses were used for assessing the prognostic significance of ZNF471. *p* values < 0.05 demonstrated a significant difference.

## Discussion

ZNF471 has been reported to be downregulated or silenced by promoter hypermethylation in gastric cancer, squamous cell carcinoma of the tongue and esophageal, and low expression of ZNF471 is associated with poor survival [24, 25, 32]. However, its functions and mechanisms in breast cancer are unclear. In the present study, we found that ZNF471 was downregulated in six of nine breast cancer cell lines and 12 of 14 pairs of breast cancer tissues. Pharmacological demethylation experiments and MSP demonstrated that its inactivation was due to promoter CpG island methylation. These data suggested ZNF471 could be a TSG in breast cancer.

We thus investigated the functions of ZNF471 in breast cancer. Previous studies have shown that KRAB-ZFPs are associated with cancer cell proliferation and are important for other biological functions, including apoptosis and migration/invasion in several tumors [21, 23, 33]. Therefore, the tumor-suppressive functions of ZNF471 in breast cancer were first investigated in this study. The results demonstrated that ectopic expression of ZNF471 in MDA-MB-231 and YCC-B1 cells significantly inhibited breast cell proliferation and colony formation, arrested the cell cycle at S phase, and promoted apoptosis. Analysis of the expression of apoptotic markers confirmed its pro-apoptotic effect: cleaved caspase 9, cleaved-PARP, Bax, and p53 were upregulated while Bcl-2 was downregulated in cells overexpressing ZNF471. In contrast, knockdown of ZNF471 in BT549 cells promoted cell growth and inhibit apoptosis. In vivo, ZNF471 also inhibited the formation of tumor xenografts in nude mice.

A significant decrease in cell migration and invasion ability was also observed after ZNF471 transfection. We found that the highly aggressive cell morphology was replaced by a less invasive morphology in ZNF471-overexpressing cells. The expression of the EMT markers Vimentin, Snail, Slug, and N-cadherin was decreased and the expression of E-cadherin was increased in ZNF471-overexpressing cells. While knockdown of ZNF471 upregulated the expression of Vimentin, Snail, Slug, and N-cadherin. Thus, part of the mechanism of ZNF471 inhibition of breast cancer cell metastasis was attenuating the EMT. MMPs regulate growth, apoptosis, angiogenesis, and cell stemness and are associated with tumor prognosis [34–37]. Increasing evidence has indicated that MMPs play a critical role in tumor metastasis by degrading the extracellular matrix, and inducing and maintaining the EMT [38–40]. The present study indicated that ectopic expression of ZNF471 downregulated the expression of MMP1 and MMP3, while MMP1 was upregulated after knocking down ZNF471. Taken together, ZNF471 suppressed breast cancer cell metastasis by attenuating the EMT and modulating MMPs. We next checked the stem cell markers ABCG2, BMI1, KLF4, MAD2, STAT3, CD44, MYC, NANOG2, and OCT4, the results of these experiments showed that ZNF471 could inhibit cell stemness in breast tumors.

The molecular mechanisms underlying the tumor-suppressive functions of ZNF471 in breast cancer were further analyzed. AKT and Wnt/β-catenin pathways have been reported to participate in the development of breast cancer, especially in cell proliferation and metastasis [6, 41, 42]. KRAB-ZFPs have been shown to suppress the growth of many tumors through Wnt/β-catenin and AKT signaling, including esophageal squamous cell carcinoma, colorectal cancer, and breast cancer [21, 33, 43]. Whether or not ZNF471 could modulate AKT and Wnt/β-catenin signaling in breast cancer was still unclear. We found that overexpressing ZNF471 downregulated phospho-AKT, phospho-GSK-3β, active-β-catenin, and their downstream target genes (c-Myc, cyclin D1); no significant changes were found in total AKT and β-catenin in MDA-MB-231 and YCC-B1 cells. While phospho-AKT, phospho-GSK-3β, active-β-catenin, c-Myc and cyclin D1 were all regulated after knockdown of ZNF471. Thus, ZNF471 could antagonize AKT and Wnt/β-catenin signaling.

## Conclusions

In summary, ZNF471 as a TSG in breast cancer and often inactivated by promoter methylation. ZNF471 inhibited cell growth, induced apoptosis, and arrested the cell cycle; it also suppressed cell migration and invasion. ZNF471 exerted its tumor suppressor function by reducing the EMT, inhibiting cell stemness, and blocking AKT and Wnt/β-catenin signaling.

## Supplementary information

**Additional file 1: Table S1.** Univariate and multivariate Cox regression analyses of ZNF471 in BC patients.

**Additional file 2: Fig. S1.** ZNF471 promoter methylation levels in breast tumor samples were detected by MSP. U: unmethylated.

## Data Availability

The datasets used and/or analyzed during the current study are available from the corresponding author on reasonable request.

## Abbreviations

ZNF471: Zinc-finger protein 471
KRAB-ZFP: Krüppel-associated box domain zinc finger protein
RT-PCR: Semiquantitative RT-PCR
qPCR: Quantitative real-time PCR
CCK-8: Cell Counting Kit-8
MSP: Methylation-specific PCR
BGS: Bisulfite genomic sequencing
TSG: Tumor suppressor gene
Aza: 5-Aza-2′-deoxycytidine
TSA: Trichostatin A
IBC: Invasive Breast Carcinoma
IDBC: Invasive Ductal Breast Carcinoma
ILBC: Invasive Lobular Breast Carcinoma
EMT: Epithelial-mesenchymal transition
PBS: Phosphate-buffered saline
PI: Propidium iodide
DAB: Diaminobenzidine
