# Correction to: The tumor suppressor Zinc finger protein 471 suppresses breast cancer growth and metastasis through inhibiting AKT and Wnt/β‑catenin signaling

**DOI:** 10.1186/s13148-022-01237-3

**Published:** 2022-01-31

**Authors:** Chunfang Tao, Juan Luo, Jun Tang, Danfeng Zhou, Shujun Feng, Zhu Qiu, Thomas C. Putti, Tingxiu Xiang, Qiao Tao, Lili Li, Guosheng Ren

**Affiliations:** 1 Key Laboratory of Molecular Oncology and Epigenetics, The First Affiliated Hospital of Chongqing Medical University, Chongqing, China; 2 Hunan Province Key Laboratory of Tumor Cellular and Molecular Pathology, Cancer Research Institute, Hengyang School of Medicine, University of South China, Hengyang, China; 3 Department of Pathology, Yong Loo Lin School of Medicine, National University of Singapore, Singapore, Singapore; 4 Cancer Epigenetics Laboratory, Department of Clinical Oncology, State Key Laboratory of Translational Oncology, Sir YK Pao Center for Cancer and Li Ka Shing Institute of Health Sciences, The Chinese University of Hong Kong and CUHK Shenzhen Research Institute, Hong Kong, China

## Correction to: Clin Epigenetics 2020 Nov 17;12(1):173 https://doi.org/10.1186/s13148-020-00959-6

Following the publication of this article, the authors noted that images of MB231 group in Fig. 5C were misplaced by mistake. The corrected Fig. 5C is now shown in this correction. The authors confirm that the conclusions of this paper are not affected, and sincerely apologize for this error and any inconvenience that may have caused [1].

**Fig. 5:**
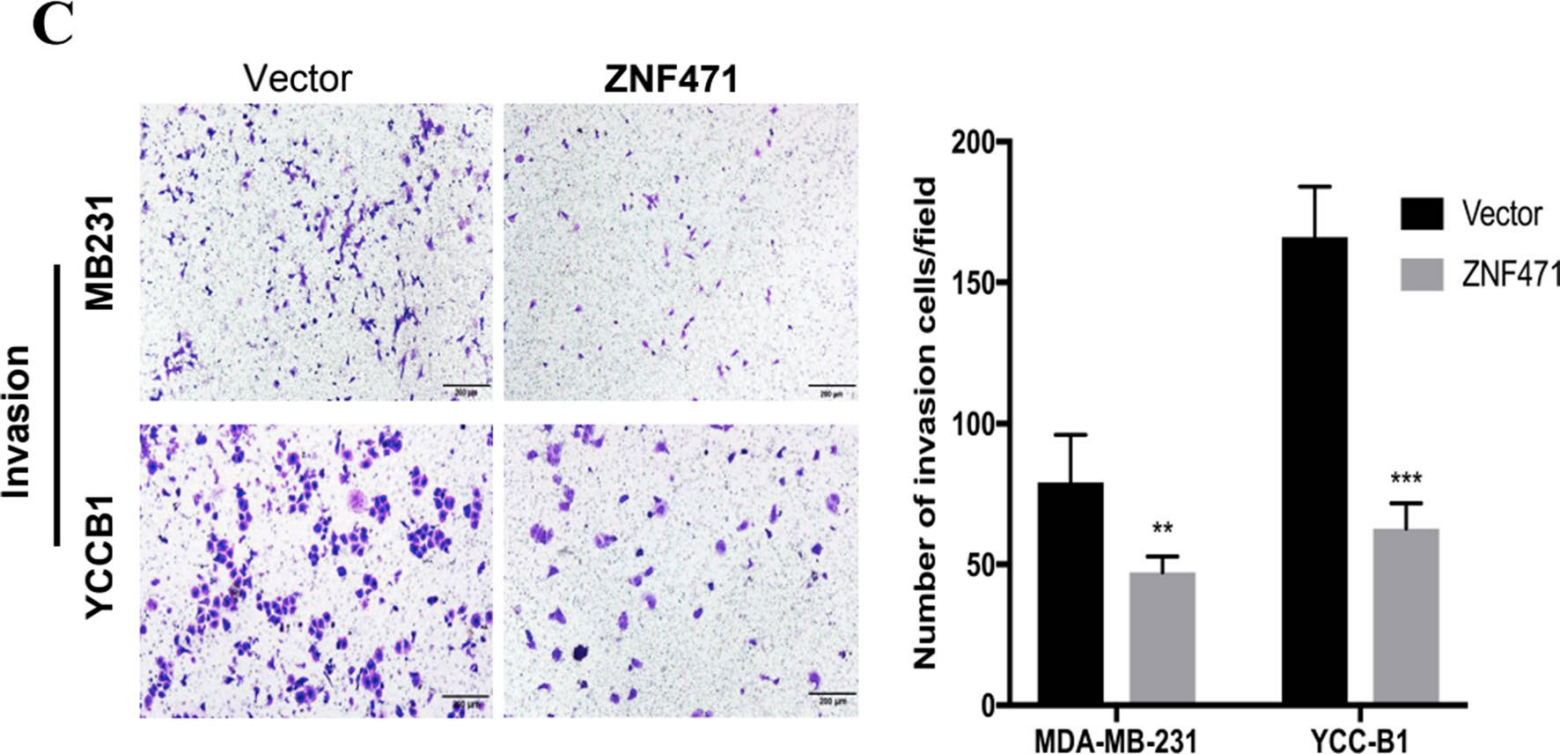
.
