# Supplementary material for: The tumor suppressor Zinc finger protein 471 suppresses breast cancer growth and metastasis through inhibiting AKT and Wnt/β-catenin signaling
Source: Clin Epigenetics. 2020 Nov 17;12:173. doi: 10.1186/s13148-020-00959-6 (PMC7672945; doi:10.1186/s13148-020-00959-6)
Supplement: Supplementary file 1 — Additional file 1: Table S1. Univariate and multivariate Cox regression analyses of ZNF471 in BC patients. [file 13148_2020_959_MOESM1_ESM.pdf]

Suppl. Table1

Univariate and multivariate Cox regression analyses of ZNF471 in BC patients.

Variants	OS			
	**Univariate analysis**		**Multivariable analysis**	
	**HR (95% CI)**	**p-value**	**HR (95% CI)**	**p-value**
**Age(≥55 vs.<55)**	0.850 (0.426-1.694	0.644		
**Lymph node(Yes vs. No)**	5.157 (1.521-17.480)	0.008*	2.711 (0.759-9.680)	0.125
**TNM stage(Ⅲ/Ⅳ vs. Ⅰ/ Ⅱ)**	2.858 (1.447-5.645)	0.003*	2.757 (1.319-5.764)	0.007*
**ER (positive vs.negative)**	0.620 (0.298-1.289)	0.201		
**PR (positive vs.negative)**	0.594 (0.296-1.191)	0.142		
**HER2 (positive vs.negative)**	0.443 (0.106-1.855)	0.265		
**ZNF471 (high vs. low)**	0.774 (0.378-1.584)	0.482		
