# Supplementary figures and images for: The tumor suppressor Zinc finger protein 471 suppresses breast cancer growth and metastasis through inhibiting AKT and Wnt/β-catenin signaling
Source: Clin Epigenetics. 2020 Nov 17;12:173. doi: 10.1186/s13148-020-00959-6 (PMC7672945; doi:10.1186/s13148-020-00959-6)

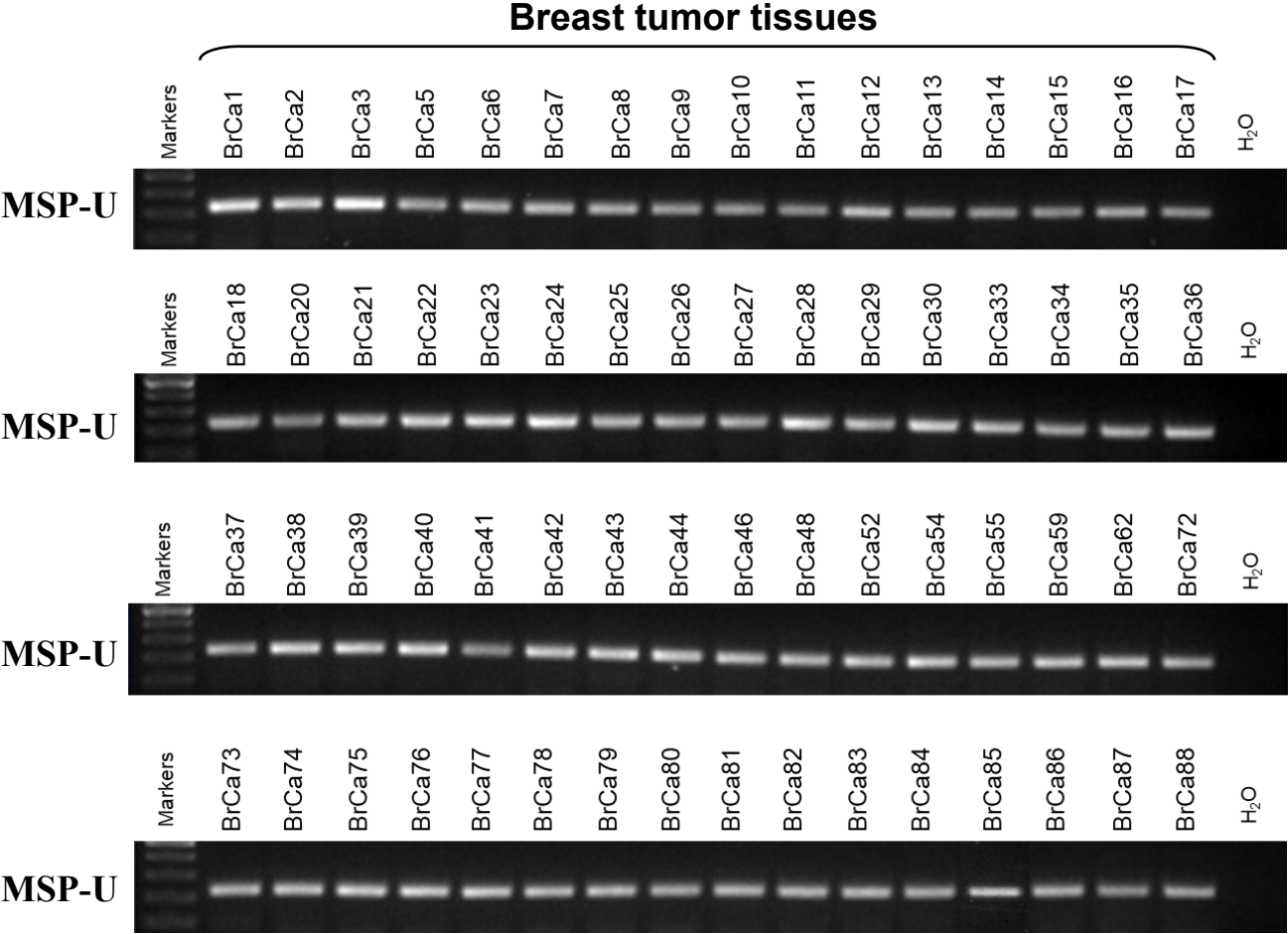

Supplement: Supplementary file 2 — Additional file 2: Fig. S1. ZNF471 promoter methylation levels in breast tumor samples were detected by MSP. U: unmethylated. [file 13148_2020_959_MOESM2_ESM.pdf]
